# Roles of Toll-Like Receptors in Nitroxidative Stress in Mammals

**DOI:** 10.3390/cells8060576

**Published:** 2019-06-12

**Authors:** Yao Li, Shou-Long Deng, Zheng-Xing Lian, Kun Yu

**Affiliations:** 1 Beijing Key Laboratory for Animal Genetic Improvement, National Engineering Laboratory for Animal Breeding, Key Laboratory of Animal Genetics and Breeding of the Ministry of Agriculture, College of Animal Science and Technology, China Agricultural University, Beijing 100193, China; yaoli19881015@126.com; 2 CAS Key Laboratory of Genome Sciences and Information, Beijing Institute of Genomics, Chinese Academy of Sciences, Beijing 100101, China; popo84350746@163.com

**Keywords:** free radicals, antimicrobial, toll-like receptors

## Abstract

Free radicals are important antimicrobial effectors that cause damage to DNA, membrane lipids, and proteins. Professional phagocytes produce reactive oxygen species (ROS) and reactive nitrogen species (RNS) that contribute towards the destruction of pathogens. Toll-like receptors (TLRs) play a fundamental role in the innate immune response and respond to conserved microbial products and endogenous molecules resulting from cellular damage to elicit an effective defense against invading pathogens, tissue injury, or cancer. In recent years, several studies have focused on how the TLR-mediated activation of innate immune cells leads to the production of pro-inflammatory factors upon pathogen invasion. Here, we review recent findings that indicate that TLRs trigger a signaling cascade that induces the production of reactive oxygen and nitrogen species.

## 1. Introduction

Reactive oxygen species (ROS) and reactive nitrogen species (RNS) are recognized for their dual role as both deleterious and beneficial species. The overproduction of ROS/RNS results in damage to cell structures, including lipids and membranes, proteins, and DNA, inhibiting their normal function. In contrast, the salutary effects of ROS/RNS occur at low/moderate concentrations in cellular responses, including in defense against infectious agents, in the function of a number of cellular signaling pathways, and the induction of a mitogenic response [1]. The subtle balance between beneficial and harmful effects of ROS/RNS is a very important aspect of living organisms and is achieved by a mechanism called “redox regulation” which maintains cellular “redox homeostasis” using the antioxidants system [2]. ROS and RNS are generated using several different processes including (i) irradiation by UV light, X-rays, and gamma-rays; (ii) as products of metal-catalyzed reactions; (iii) by neutrophils and macrophages during inflammation; and (iv) as products of mitochondria-catalyzed electron transport reactions [3]. An important potential source of oxidizing agents is the phagocytic leukocytes within the body. Neutrophils, monocytes, and macrophages are the most prominent immune cell types that release various pro- and anti-inflammatory mediators for both host defense and inflammatory responses [4]. Oxidation intermediates are also involved in these processes. The cumulative production of ROS/RNS from either endogenous or exogenous sources is termed oxidative stress. Oxidation intermediates are essential activators of oxidative stress. This is because low levels of free radicals, including ROS and RNS, form a stressful oxidative environment that can clear invading pathogens and maintain physiological homeostasis [5]. Although innate immunity is the first line of defense against pathogens, Toll-like receptors (TLRs) also play a crucial role in the early host defense mechanism [6]. In recent years, there have been a significant number of discoveries regarding how the TLR-mediated activation of innate immune cells leads to the production of pro-inflammatory factors upon pathogen invasion. In this review, we discuss recent research findings on TLR activities, with a particular focus on TLR-mediated signaling pathways that are activated during nitroxidative stress.

## 2. Nitroxidative Stress

### 2.1. Formation of Nitroxidative Stress

Nitroxidative stress is a cellular condition that reflects a physiological imbalance where excessive reactive nitrogen and reactive oxygen species are present. Under this condition, an excessive production of ROS and RNS occurs, exceeding a level that the body’s antioxidant mechanisms can cope with. Examples of ROS species include the superoxide anion (O_2_^●^^−^), hydrogen peroxide (H_2_O_2_), and hydroxyl radicals (•OH) [7]. RNS include nitric oxide (NO^●^), nitrogen dioxide (NO_2_), and the powerful oxidant peroxynitrite (ONOO^−^) [8]. During microbial infections, excessive NO^●^ produced has varying functions, ranging from anti-microbial and anti-inflammatory host defense and cell protection to proinflammatory and cytotoxic activities [9]. The NO^●^ produced in inflamed tissues during infection or inflammation are affected by the concomitant production of oxygen radicals, particularly O_2_^●^^−^ and H_2_O_2_. The interaction of NO^●^ with reactive oxygen species causes the formation of several reactive nitrogen oxides, these reactive nitrogen intermediates have a great possibility of causing oxidative and nitrative stress through the oxidation and nitration of biological molecules [10]. A study in mice shows that O_2_^●^^−^ was produced by neutrophils and macrophages in the liver, which is associated with prolonged granulomatous lesions in infected with *Salmonella typhimurium*. The inhibition of O_2_^●^^−^ generation by in vivo superoxide dismutase (SOD) treatment results in a reduction in the area of liver lesions, simultaneously accelerating bacterial growth in the liver. The result suggests that O_2_^●^^−^ may be involved in the host defense mechanism against *S. typhimurium* infections [11]. Granulocyte peroxidases, such as myeloperoxidase, play an important role in oxidative stress [12]. In neutrophils, H_2_O_2_ produced by O_2_^●^^−^ is metabolized by myeloperoxidase into a strong oxidant, hypochlorous acid (HOCl), in the presence of chloride ions. HOCl plays an important role in host defense and inflammatory tissue injury. In addition, myeloperoxidase generates reactive nitrogen species in vivo only when nitrite and nitrate are available [13]. Overproduced ROS and RNS can easily and rapidly react with intracellular macromolecules, causing oxidative damage to cellular structures that result in progressive physiological dysfunction, which is associated with many pathological conditions. ROS are the major causative factor in steatohepatitis and insulin resistance [14]. Various neurodegenerative diseases, such as Parkinson’s disease (PD), Alzheimer’s disease (AD), Huntington’s disease (HD), and amyotrophic lateral sclerosis (ALS), can be the result of oxidative stress [15].

### 2.2. Antioxidant Systems

There are two types of antioxidant systems in the body. The first one is formed by antioxidant enzymes, which includes superoxide dismutase (SOD), catalase (CAT), and glutathione peroxidase (GSH-Px). The other is formed by non-enzymatic antioxidants which includes vitamin C, glutathione (GSH), melatonin, and trace elements [16]. Antioxidant substances catalyze the formation of active oxygen intermediates, scavenge free radicals, and terminate peroxidation. ROS is mainly produced by Nicotine adenine dinucleotide phosphate (NADPH) oxidase [17]. When stimulated, NADPH oxidase induces electron transmembrane transport and generates O_2_^●^^−^. As O_2_^●^^−^ is unstable, it is rapidly converted into H_2_O_2_, which can then be transformed into H_2_O and O_2_^•−^ or other active oxide derivatives.

RNS family members are derived from NO^●^ through the action of nitric oxide synthase (NOS). NOS is a multidomain enzyme that contains binding sites for the cofactors NADPH, flavin adenine dinucleotide (FAD), flavin mononucleotide (FMN), and (6*R*-)5,6,7,8-tetrahydrobiopterin (BH_4_). It catalyzes the production of NO^●^ and L-citrulline from L-arginine. The availability of L-arginine is one of the rate-limiting factors in cellular NO^●^ synthesis and secretion. There are three members of the NOS family: neuronal NOS (nNOS), endothelial NOS (eNOS), and inducible NOS (iNOS). As nNOS and eNOS are constitutively expressed and not inducible, they are not associated with the inflammatory response [18]. The iNOS isoform is upregulated by inflammatory stimulating factors [19] and can be activated by phagocytes after stimulation with PRR agonists, IFN-γ, and proinflammatory cytokines [20]. In turn, the produced NO^●^ is able to stimulate the expression of the transcription factor NF-E2-related factor-2 (Nrf2) in macrophages, upregulate ferroportin (FPN), and contribute to nutritional immunity in macrophages [21]. Nrf2 plays a key role in anti-inflammation and oxidation stress and triggers the expression of the downstream target genes catalase (CAT), superoxide dismutase (SOD), glutathione S-transferase alpha 1 (GSTα1), quinone oxidoreductase 1 (NQO1), γ-glutamylcysteine synthetase (γ-GCS), and heme oxygenase-1 (HO-1).

Phosphoinositide 3-kinase (PI3K) and MAPK signaling pathways regulate activation of the Nrf2-antioxidative response element pathway [22]. Oxidative stress activates NF-κB and induces inflammatory responses. Together, Nrf2 and NF-κB cooperatively regulate the oxidative stress response [23]. Oxidation intermediates exert antimicrobial actions against a broad range of pathogens. Indeed, chronic granulomatous disease (CGD) patients that are deficient in oxidation intermediates are susceptible to extracellular bacterial infections [24]. CGD is characterized by inherited defects in the innate immune system resulting from mutations in the genes encoding any of the five components of the NADPH oxidase complex, including gp91-phox, p22-phox, p40-phox, p47-phox, and p67-phox [25]. However, some bacteria have devised strategies to escape killing using oxidation intermediates. *Mycobacterium tuberculosis* binds to macrophage-expressed complement receptor 1 (CR1) and complement receptor 3 (CR3) to overcome reactive oxygen intermediates (ROI) and reactive nitrogen intermediates (RNI) production. This allows for the safe access of pathogens to their intracellular habitat. *Staphylococcus aureus* releases adenosine to overcome oxidative killing in phagocytes and produces the enzymes SOD and CAT to eliminate oxidation intermediates [26]. Mitochondria are major sources of ROS production in cells [27]. Mice with reduced macrophage ROS levels (through the overexpression of CAT in their mitochondria) showed an impaired ability to kill intracellular bacteria following intraperitoneal infection with *Salmonella typhimurium*. This confirms that ROS play a key role in bactericidal activity [28].

## 3. TLRs and Their Signaling Pathway

### 3.1. An Introduction on TLRs

Microbial structures and molecules are crucial for their survival and virulence. Pathogen-associated molecular patterns (PAMPs) are highly conserved structural components that are uniquely associated with microorganisms [5]. These include LPS, lipoproteins, carbohydrates, flagellin, and nucleic acids. Upon long-term exposure to pathogens, the innate immune system provides a faithful mechanism to rapidly sense and respond to PAMPs using a number of structurally unrelated host proteins called pattern recognition receptors (PRRs) [6]. PRRs play important intracellular roles to eliminate infection and initiate inflammatory responses [29]. To date, four different classes of PRR families have been identified, including C-type lectin receptors (CLRs), RIG-I-like receptors (RLRs), nucleotide-binding-domain and leucine-rich-repeat-containing receptors (NLRs), and TLRs [30]. PRRs are strategically distributed in the cell to enable the recognition of both extracellular and intracellular pathogens. For example, TLRs and CLRs localize to plasma or endosomal membranes, whereas RLRs and NLRs are cytosolic [31]. TLRs are type I integral membrane glycoproteins that are characterized by an N-terminal extracellular domain with leucine-rich repeats (LRRs) that recognize PAMPs, a transmembrane region, and a cytoplasmic Toll/IL-1R homology (TIR) domain that mediates downstream signaling [30]. TLRs are one of the most-investigated PRR families and are able to detect a wide range of PAMPs, including bacterial lipopolysaccharides (LPS), lipoproteins, flagellin, and nucleic acids. Many structural studies on TLRs, including X-ray crystallography, have been undertaken to investigate the basis of their ligand recognition [5]. In zebrafish (Danio rerio), 19 putative TLR variants, the orthologs of mammalian TLR 2–5 and 7–9, a fish specific receptor type group, and three putative splice variants have been identified [32]. Some studies on oxidative stress in the zebrafish model have been done, including a study evaluating the effects of dietary supplementation with the probiotic *Bacillus amyloliquefaciens* R8, which has a heterologous expression of xylanase from rumen fungi, on zebrafish. The result shows it improved expression levels of oxidative stress-related genes in the fish liver [33]. Zebrafish embryo exposure to Triclocarban (TCC) at environmental concentrations significantly affects the expression of immune-response-related genes following oxidative stress and the release of proinflammatory mediators through the Toll-like receptor signaling pathway [34]. Thirteen TLRs have been discovered in mammals to date [35]. TLR1-9 is present in both humans and mice. Mouse TLR10 is nonfunctional owing to a retroviral insertion. Human TLR11 is a pseudogene, and TLR12 and TLR13 are absent from the human genome. Studies with TLR-knockout mice have demonstrated that each TLR subgroup recognizes distinct PAMPs and initiates a particular immune response (Table 1) [36]. Studies of *Drosophila* TLRs also support the fact that distinct TLRs may differ in their signaling ability. In *Drosophila*, *toll* and *18-wheeler* sharing homologous cytoplasmic domains activate different and nonoverlapping gene expressions when faced with fungi and bacteria. TLRs are expressed in a variety of cells and tissues, including dendritic cells, mononuclear macrophages, and granulocytes. Immune cells are capable of recognizing distinct molecular patterns to elicit a specific response against pathogens or endogenous factors released as a result of cellular damage. TLRs can recognize PAMPs in different cell compartments, including the plasma membrane, endosomes, lysosomes, and endocytic lysosomes. However, the TLRs must be appropriately localized in cells to recognize their ligands. This is also important for self-tolerance and downstream signal transduction. When TLR4 interacts with myeloid differentiation factor 2 (MD2) and CD14, an LPS receptor is then combined with a ligand [37]. This alerts the immune system to the presence of invading organisms, so that an immediate response can be configured to contain the pathogen. Later, during the start of acquired immunity, the recognition event is provided information about the nature of the invading microorganism. From this information, it is determined whether T helper (Th) 1 cells differentiate into Th1 cells that promote cell-mediated immunity or Th2 cells that promote humoral responses [38].

### 3.2. Signaling Pathway of TLRs

TLRs generally function as homodimers. However, TLR2 exists as a heterodimer with another TLR molecule, for example TLR2/TLR1 and TLR2/TLR6. TLR2 recognizes lipoteichoic acid, lipopeptides, peptidoglycan, and Zymosan [41], whereas TLR4 homodimers specifically recognizes lipopolysaccharides (LPS) from Gram-negative bacteria heat-shock proteins [42] and viral components such as the fusion protein from respiratory syncytial virus (RSV) [24]. TLR5, TLR9, and TLR3 recognize the flagellin component of bacterial flagella, genomic DNA, and the viral replication intermediate dsRNA, respectively [43,44,45,46]. All TLRs except TLR3 utilize MyD88 to activate NF-κB and MAPK and induce an overproduction of inflammatory cytokines [47]. This pathway is called the MYD88-dependent pathway. Toll-interleukin1 receptor (TIR) domain containing adaptor protein (TIRAP) assists the recruitment of MyD88 to the surfaces of TLR2 and TLR4. TIRAP has a lipid-binding region that binds to PI (4,5) P in the plasma membrane and PI (3) P on the endosome, allowing the formation of functional signaling complexes at their respective positions [48]. In MYD88-independent pathways, TRIF is recruited to TLR3 and TLR4, which results in the activation of interferon regulatory factor 3 (IRF3), nuclear factor kappa-light-enhancer of activated B cells (NF-κB), and mitogen-activated protein kinase (MAPK) to induce the production of type I interferons and inflammatory cytokines. TRAM is selectively recruited to TLR4 to link TRIF and TLR4. Although TLR3 is able to bind directly to TRIF, this interaction requires the phosphorylation of two tyrosine residues in the cytoplasmic region of TLR3 [49,50].

**Figure 1:**
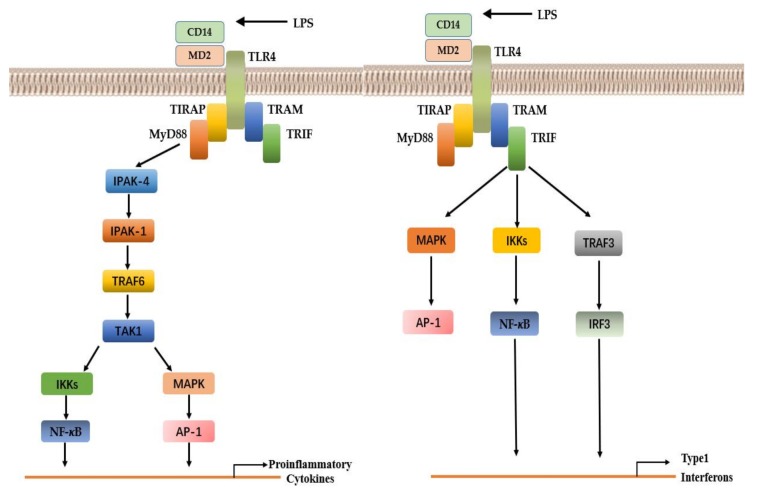
MYD88-dependent pathway (left). MYD88-independent pathways (right) [52].

Two distinct TLR signaling pathways have been identified based on the availability of adaptor molecules. Both pathways activate downstream signaling molecules that lead to the secretion of inflammatory cytokines, type I interferon (IFN), chemokines, and antimicrobial peptides [51]. These instigate neutrophil recruitment, macrophage activation, and the induction of IFN-related genes, which directly kill pathogens. In addition, the activation of the TLR signaling pathway leads to the maturation of dendritic cells (DCs) and contributes to adaptive immunity (Figure 1).

## 4. TLRs and Nitroxidative Stress

Under normal conditions, TLRs are maintained at relatively stable levels by intracellular modulation (such as alternative splicing) and degradation by ubiquitination and deubiquitination. Transgenic animals overexpressing TLRs and TLR knockouts have been studied to determine the role of TLRs during bacterial infection in vivo. *S. typhimurium* is a Gram-negative bacterium that replicates in macrophages and has PAMPs that are detectable by at least four TLRs: lipoprotein (TLR2), LPS (TLR4), flagellin (TLR5), and CpG-DNA (TLR9) [53]. In this review, we will focus on data obtained for the TLR2 and TLR4 signaling pathways in mammalian immune cells, which are linked to nitroxidative stress.

Molecules found in nitroxidative stress, such ROS and NO^●^, are found in response to microbial invasion during the neutrophil and macrophage respiratory burst. Both oxidative stress and infective stress can share the same TLR signaling pathways [54]. TLRs interact with adaptor molecules such as MyD88 and TRIF to activate downstream signaling through NF-κB, AP-1, and interferon-regulatory factor-3 (IRF-3). The expression of inflammatory mediators is upregulated; involve notably pro-oxidant enzymes such as NOX and iNOS, producing high levels of ROS [55]. To examine whether TLR signaling could enhance ROS production, a study in RAW 264.7 macrophages shows the use of all kinds of TLRs agonist. The production of ROS was triggered only upon signaling from the cell-surface TLRs (TLR1, TLR2 and TLR4), the same as exposure of cells to rotenone and antimycin A, compounds known to increase mitochondrial O_2_^●–^ generation, and increased cellular H_2_O_2_ [28].

### 4.1. TLR2 and Nitroxidative Stress

TLR2 recognizes Gram-positive bacteria, including mycobacteria, and viruses and their products. Most Gram-positive bacteria, such as *Bacillus anthracis*, *S. aureus*, and *Clostridium tetani,* cause a wide range of diseases in both immunocompetent and immunocompromised hosts [43]. TLR2 is widely expressed in innate immune cells and epithelial cells and is highly expressed in peripheral blood monocytes. TLR2 was found to induce neutrophil activation and its expression is upregulated when it recognizes its corresponding PAMP. This results in the production of NO^●^ and pro-inflammatory cytokines, including tumor necrosis factor α (TNF-α), interleukin (IL)-1β, IL-6, and chemokines. TNF-α and IL-1β are both able to stimulate the production of monocyte chemotactic protein-1 (MCP-1). MCP-1 expression is also induced by oxidative stress [56]. TLR-2 relies on the MyD88 signaling pathway to activate the NF-κB and MAPK pathways. Phosphorylated MAPK then activates the transcription factor activator protein-1 (AP-1) and the PI3K/protein kinase B (Akt) signaling pathway to induce immune responses. AP-1 is a transcription factor that mediates pro-inflammation through core components including c-Jun and c-Fos. Overexpression of c-Jun induces the production of inflammatory mediators [57].

During pathogen infection, inflammation is accompanied by an anti-inflammation reaction. Native CD4^+^ cells differentiate into Th1 and Th2 cells. The Th1 response produces IL-12 to promote the production of IFN-γ, which functions to inhibit the differentiation of Th2 cells. The Th2 response is characterized by low IFN-γ levels and high IL-4 levels, as well as the production of IL-6 and IL-10. IL-6 produced by Th2 cells acts as a mediator of the acute phase response. It is able to accelerate the infiltration of inflammatory cells [58] but, at later phases, can increase anti-inflammatory cytokine expression to prevent an immune overreaction [59]. IFN-γ levels are low during the Th2 response as it is able to inhibit Th2 cells and the production of IL-10 to decrease the levels of Th1-related cytokines. Mice deficient in TLR2 experienced strong immune reactions and were susceptible to *S. aureus* [60]. Another study revealed that TLR2 signaling contributes to high mortality during polymicrobial intra-abdominal sepsis. This suggests that a novel therapeutic approach to treat severe sepsis would be to specifically target the TLR2 signaling pathway [61].

A previous study reported that mice challenged with Pam3CSK4, a TLR2 agonist, had reduced infiltration of chemokines and inflammatory cells within their tissues [62]. Furthermore, a study by our laboratory revealed that Pam3CSK4 could activate monocytes/macrophages. An overexpression of TLR2 in transgenic goats caused early expression of the pro-inflammatory cytokines’ TNF-α and IL-1β, followed by a continuous increase in the expression of the anti-inflammatory factor IL-4. In addition, TLR2 overexpression induced the expression of Th1 type cytokines. A skin inflammation experiment showed that TLR2 overexpression accelerated the inflammatory process in transgenic goats [63].

Free radicals are necessary for the host defense against microbial invasion and inflammatory injury. Following macrophage activation by TLR2, high levels of NOS and ROS are released, in addition to NO^●^ and H_2_O_2_, to the area of inflammation under oxidative stress. Intriguingly, a study using RAW264.7 cells indicated that NF-κB activation was significantly enhanced upon exposure to NO [64]. Even relatively low levels of NO^●^ are able to trigger downstream pathways and maintain the correct functioning of the defense mechanism. However, NF-κB activation is inhibited by high concentrations of NO^●^ or the overexpression of antioxidants. The inhibition of NF-κB activation has been shown to decrease the inflammatory response and prevent tissue damage. GSH, SOD, and CAT are key anti-oxidative substances in a host organism. Activated NF-κB can induce the secretion of pro-inflammatory factors and also reduce cellular SOD activity. As described earlier, NO^●^ levels are regulated by iNOS. Activated Nrf2 can induce the expression of the proinflammatory gene COX-2 by inhibiting NF-κB activity, which reduces iNOS expression [65]. Furthermore, Nrf2 activation can directly regulate c-Jun signaling activity and suppress COX-2 expression. COX-2 overexpression was found to inhibit PI3K activity and the Nrf2-mediated anti-oxidation reaction [30]. A study of healthy people and coronary artery disease (CAD) patients shows the activation of the Nrf2 pathway as an antioxidant response mechanism in monocyte-derived macrophages (MDMs) [66]. The activation of Nrf2 protects human coronary artery endothelial cells against oxidative challenge [67].

**Figure 2:**
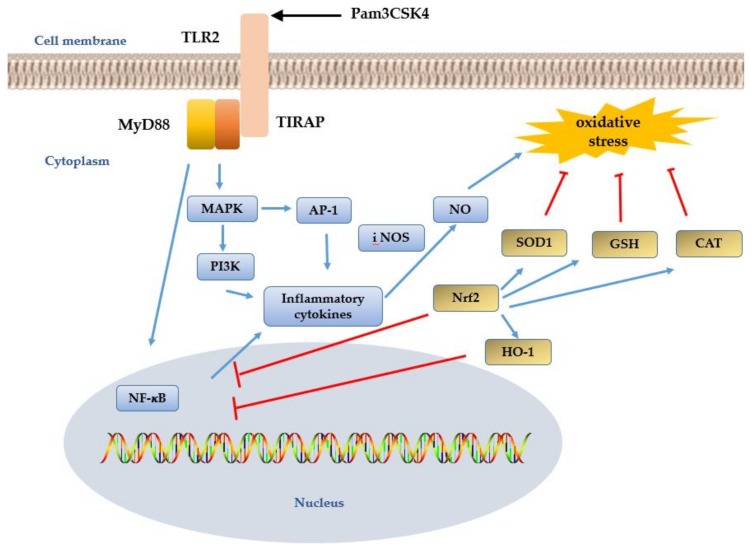
The TLR2 signal pathway involved in oxidative stress [48,55]. The TLR2 signaling pathway activates the MYD88 pathway to activate the NF-κB and MAPK pathways under the action of the ligand Pam3CSK4. Phosphorylation of MAPK then activates transcription factor activator protein-1 (AP-1) and PI3K/protein kinase signaling pathways to induce an immune response. AP-1 is a transcription factor that mediates pro-inflammatory factors, and NO^●^ levels are regulated by iNOS. Nrf2 can induce the expression of pro-inflammatory factors by inhibiting the expression of pro-inflammatory NF-κB, which is a rate-limiting enzyme involved in the anti-inflammatory reaction and can induce oxidative stress. GSH, SOD, and CAT are all in a key antioxidant in the host organism. Activated NF-κB reduces cellular SOD activity.

AP-1 family members include Jun and Fos. Jun subclasses include c-jun and JunB. Fos subclasses include c-fos and FosB. Different types of AP-1 transcription factor dimer combinations have different functions in gene expression regulation. Increased levels of c-Fos and c-Jun subunits negatively regulate the expression of the anti-oxidation genes NAD(P)H: quinone oxidoreductase 1 (NQO1), GSTα1, SOD1, and CAT. HO-1 is a rate-limiting enzyme that participates in anti-inflammatory reactions and is induced in response to oxidative stress. HO-1 gene activation has been demonstrated to inhibit AP-1 activity [68]. The activity of AP-1 was observed to increase in Nrf2 knockout cells throughout the JNK/c-Jun pathway, leading to the upregulation of HO-1 expression [69]. Our laboratory results indicate that Nrf2 upregulation, through the overexpression of TLR2 in transgenic goats, inhibited COX-2 expression and increased the expression of the c-Jun gene in monocytes/macrophages by TLR2 ligand stimulation. In addition, lower levels of anti-oxidation stress enzymes were observed in cells overexpressing TLR2 compared with wild-type cells. This, in turn, improved the activity of GSH, and the GSH consumed could be rapidly resynthesized [63]. TLR2 overexpression also upregulates PI3K and increases HO-1 gene expression. However, the concentrations of malondialdehyde (MDA), a marker of lipid peroxidation, and NO^●^ in cells overexpressing TLR2 remained relatively low and were maintained at stable levels (Figure 2). In combination, these data indicate that tissue damage can be prevented through TLR2 overexpression.

### 4.2. TLR4 and Nitroxidative Stress

A specific ligand is mediated by the TLR4/MD2 complex, together with the co-receptor CD14, then recruits downstream adaptors to activate the MyD88- and TRIF-dependent pathways [58]. By activating these two pathways, TLR4 participates in innate immunity in the host’s defense against Gram-negative bacterial infections, as well as in many inflammatory and autoimmune diseases. The TLR4 signaling pathway is activated upon the invasion of animal cells by pathogenic microorganisms. This triggers a cascade of reactions to promote the production and release of inflammatory cytokines, which induces the chemotactic aggregation of macrophages. However, recently, an interesting study has demonstrated palmitate-stimulated CD11b + F4/80 low hepatic infiltrating macrophage ROS generation by dynamin-mediated endocytosis of TLR4 and NOX2, independent from MyD88 and TRIF [14].

Several studies have demonstrated that TLR4-mutant enterocytes have a decreased sensitivity to LPS compared with wild-type cells [70]. Moreover, TLR4-mutant mice exhibit a suppressed inflammatory response [71]. This is because TLR4-deficient mice are unable to secrete IL-1 or IL-12 and express IL-6 at lower levels when stimulated with LPS [72]. Mice with targeted deletions of multiple inflammatory immune and antioxidant genes are susceptible to oxidative lung injuries [73]. Overexpression of TLR4 in mice amplifies the host response to LPS and provides a survival advantage of increased disease resistance [74]. LPS recognition stimulates TLR4 signaling pathways that lead to the activation of multiple downstream signaling pathways. In research on TLR4 overexpressing ovine macrophages, TLR4 initially promoted the production of proinflammatory cytokines TNFα and IL-6 by activating TLR4-mediated IRAK4-dependent NF-κB and MAPK (JNK and ERK1/2) signaling. This was later impaired due to the increased internalization of TLR4 into the endosomal compartment of macrophages. Then, the overexpression of TLR4 triggered TBK1-dependent interferon-regulatory factor-3 (IRF-3) expression, leading to the induction of IFN-β and IFN-inducible genes. The bacterial burden after infection with live *S. typhimurium* in these macrophages was decreased significantly [75].

TLR4 has also been shown to be involved in the phagocytosis of various bacterial species via its interaction with MAPK, Janus kinase 2 (Jak2), PI3K, and various receptors [76]. The expression of these factors was observed to increase following *S. typhimurium* infection of transgenic TLR4-overexpressing sheep. Ovine monocytes/macrophages overexpressing TLR4 were able to phagocytize higher numbers of bacteria and also showed a higher phagocytic ability at an early stage, even when infected with a lower bacterial dose. This suggests that TLR4 overexpression causes an increase in scavenger receptor expression. In addition, TLR4 overexpression increased the number of bacteria that could adhere to individual monocytes/macrophages, as well as the number of monocytes/macrophages able to participate in bacterial adhesion. In contrast, the inhibition of TLR4 reduced *S. typhimurium* internalization, actin polymerization, scavenger receptor expression, and the adhesive capacity of immunocytes. These findings are consistent with previous data using PI3K inhibitors, suggesting that TLR4 interacts with PI3K to enable *S. typhimurium* phagocytosis through the regulation of scavenger receptor expression, actin polymerization, and the alteration of the adhesive capacity of monocytes/macrophages [77]. In the infection experiment of *Escherichia coli* in a TLR4-overexpressing transgenic (Tg) sheep model, monocytes of Tg sheep could phagocytize more bacteria and exhibited higher adhesive capacity. Using specific inhibition of p38 MAPK, c-Jun N-terminal kinase (JNK), or extracellular signal-regulated kinases (ERKs), the TLR4-dependent *E. coli* internalization in sheep monocytes was reduced. p38, JNK, and ERK are all mitogen-activated protein kinases (MAPKs), which are important downstream signaling molecules of TLR4. These results provide valuable insight into the bacterial internalization mechanisms in sheep [78].

Our research laboratory generated transgenic sheep overexpressing TLR4 that had two TLR4 copies inserted into germ cells. About 90–95% of Gram-negative bacteria are considered to be harmful to their hosts. Many intracellular bacteria, such as *Brucella*, *Tubercle bacilli*, and *Salmonella*, are pathogenic to both animals and humans and they can be transmitted from animals to humans. As China has a large sheep breeding industry, the most harmful of these infections are brucellosis caused by *Brucella melitensis*. It not only brings huge economic losses but also causes security risks. The design of TLR4 expression sheep provides an important theoretical basis for transgenic disease-resistant breeding. LPS stimulation of these transgenic sheep enhanced the inflammatory response, which aids the clearance of pathogens. This could cause excessive oxidative stress that would cause tissue damage [79]. However, TLR4 is able to tightly regulate oxidative stress throughout these processes. Upon bacterial invasion, TLR4 triggers the activation of inflammatory factors such as NF-κB and AP-1. The mechanism by which TLR4 mediates the production of ROS involves the membrane-associated enzyme complex NADPH oxidase. Mouse peritoneal macrophages activated by LPS resulted in an increase in the functional activity of NADPH oxidase [80]. The NADPH oxidase inhibitor, apocynin, protected mice from LPS-induced lethality by decreasing the expression level of inflammatory cytokines in vivo [81]. Research using a yeast two-hybrid and glutathione S-transferase pull-down assay model has suggested that there may be a direct interaction between TLR4 and NADPH oxidase in mediating the LPS-induced production of ROS. The carboxy-terminal region of Nox4, a subunit of NADPH oxidase, interacted directly with the Toll/IL-1 receptor (TIR)-domain of TLR4 after LPS stimulation [82]. Intriguingly, another study on neutrophils shows that the synthesis of NADPH oxidase was also controlled by TLR4 through the interleukin-1 receptor-associated kinase 4 (IRAK4) pathways. Phosphorylation of the cytosolic factor p47 phox is essential for the activation of NADPH oxidase. The data shows that p47 phox is a substrate for IRAK-4 [83].

**Figure 3:**
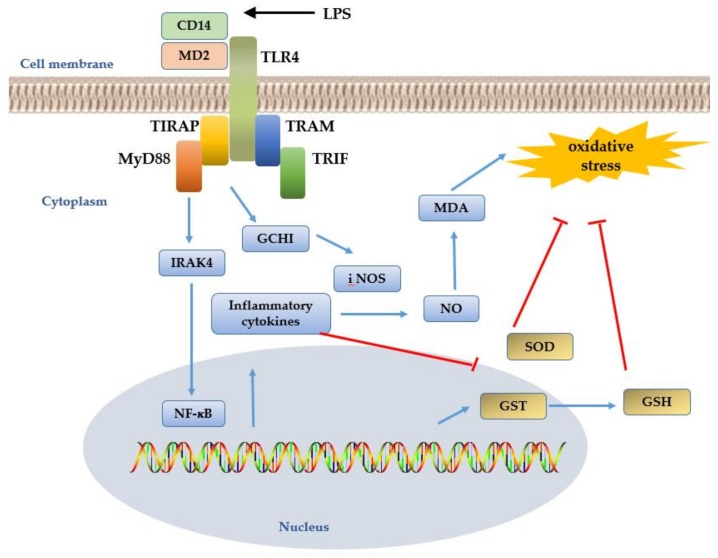
TLR4 signal pathway in oxidative stress [73,75]. TLR4 interacts with myeloid differentiation factor 2 (MD2), CD14, and specific ligand LPS receptors, then recruits downstream adaptors to activate MyD88− and TRIF-dependent pathway-mediated IRAK4-dependent NF-κB, which can trigger the transcription-promoting NO^●^. The GCHI-iNOS gene shows a significant increase in the expression of iNOS and NO, which accelerates the inflammatory response by reducing SOD activity and increasing MDA production. GSH eliminates free radicals and prevents oxidative damage.

In addition, TLR4 can trigger the transcription of the iNOS gene, which promotes NO^●^ production. Furthermore, iNOS produces peroxide and O_2_^●−^ radicals. NO^●^ synthesis not only requires L-arginine as a substrate but also several cofactors for its catalytic activity [84]. BH4 is an essential co-factor for all NOSs. BH4 bioavailability is a critical factor in regulating the balance between NO^●^ and O_2_^●−^ production [85]. This has been demonstrated when diabetic mice underwent transgenic over-induction of BH4 synthesis to preserve NO^●^-mediated endothelial function [86]. ROS such as O_2_^●−^ and ONOO^−^ are able to rapidly oxidize BH4, leading to BH4 catabolism and depletion [87]. The oxidation of BH4 results in the formation of dihydrobiopterin (BH2), which binds to NOS and generates O_2_^●–^, but not NO^●^ [88]. Guanosine triphosphate cyclohydrolase I (GCHI) is a rate-limiting enzyme in BH4 synthesis. A novel study investigating the relationship between activated GCHI with BH4 revealed that GCHI-transgenic mice stimulated with LPS showed a marked increase in the expression of renal iNOS and NO^●^ production (Figure 3) [89].

ROS can induce NF-κB activation and upregulate the cytokine-induced iNOS gene, resulting in the release of excessive amounts of NO^●^. Our research revealed that these inflammatory factors accelerate the inflammatory response by reducing SOD activity and increasing MDA production. SOD is depleted during the clearance of oxygen free radicals. Therefore, tissues can then be subsequently damaged by ROS accumulation [90]. Inflammatory events are often accompanied by oxidative stress, which generates lipid peroxidation products such as 4-hydroxy-2-nonenal (4-HNE). The study in primary neuronal cultures from TLR4 mutant mice and wild-type control mice show that TLR4 expression increases in neurons when exposed to the HNE, and TLR4 signaling increases the vulnerability of neurons to oxidative stress. This indicates that TLR4 signaling may play a role in Alzheimer’s disease (AD) pathogenesis, possibly being activated by membrane-associated oxidative stress [91]. Another study found that 4-HNE blocks TLR4-mediated macrophage activation, gene expression, and phagocytic functions. This is done at least partly by suppressing receptor dimerization [92]. The expression of the anti-inflammatory enzyme HO-1 was found to increase in sheep overexpressing TLR4, which can directly regulate AP-1 expression. Increased HO-1 activity can suppress TLR4-induced signal transduction [93]. Our data showed that CAT activity was reduced and GSH-Px expression was increased following the LPS stimulation of sheep overexpressing TLR4. Another study suggested that GSH-Px acts in the place of CAT to eliminate H_2_O_2_ in certain tissues. We predict that GST may play a critical role in regulating antioxidative enzyme expression. GST transcription can be upregulated and downregulated with AP-1 and GSH, respectively. ROS-mediated inflammation can be reversed by adding exogenous GSH, which can be transferred to glutathione disulfide (GSSG) to eliminate free radicals and avoid oxidative damage to tissues. The overexpression of TLR4 in sheep resulted in a more rapid GSH consumption that dramatically increased GSSG and resulted in severe oxidative damage. Our laboratory showed that TLR4-induced oxidative stress occurred as a result of NO^●^ synthesis.

TLR4 and its downstream signaling pathways are involved in the activation of GCHI expression [94]. In turn, GCHI plays an important role in the regulation of iNOS expression. The expression of NADPH oxidase and iNOS in a group of transgenic sheep overexpressing TLR4 was significantly greater compared with a WT group at 1 and 8 h, but these returned to normal levels at 48 h. This observation indicates that TLR4 can regulate the expression of both NADPH oxidase and iNOS. In addition, overexpressed TLR4 inhibited SOD activity and triggered AP-1 to activate downstream antioxidative genes that protect against oxidative stress [90].

## 5. Conclusions

Toll-like receptors (TLRs) act as immune receptors to initiate innate immunity and acquired immunity. They play an important role in the development of oxidative stress in the body. TLRs activate cells to produce pro-inflammatory factors in pathogen invasion, which can act as secondary messengers to regulate oxidative stress. Furthermore, TLRs can produce antioxidant mechanisms that interact to regulate oxidative stress. Our study shows that under the stimulation of LPS, transgenic overexpressing TLR4 sheep can rapidly trigger the TLR4 signaling pathway and upregulate the expression of cytokines in a short time, thus reducing the inflammatory reaction time, which is of great significance for improving the disease resistance of sheep. Positive individuals can significantly increase the adhesion of bacteria, which plays an important role in the timely removal of pathogens. Next, we will further evaluate the phagosome clearance ability of our transgenic sheep during an infection of phagocytic bacteria.

## Figures and Tables

**Table 1:** TLR Recognition of Microbial Components [36,39,40].

TLR Usage	Expression Patterns in Leucocytes	Cellular Localization	Microbial Component Recognized by the Receptor
TLR1	T-Lymphocytes	Cell surface	Triacyl lipopeptides
	B-Lymphocytes		
	Natural killer cells		
	PMNs		
	Mononuclear phagocytes		
	Dendritic cells (DCs)		
TLR2	T-Lymphocytes	Cell surface	Triacyl lipopeptides
	PMNs		Diacyl lipopeptides
	Mononuclear phagocytes		Lipoteichoic acid
	DCs		Peptidoglycan
			Porins
			Lipoarabinomannan
			Phospholipomannan
			Glucuronoxylomannan
			tGPI-mutin
			Hemagglutinin protein
			Not determined
			Zymosan
TLR3	DCs	Endosome	dsRNA
TLR4	PMNs	Cell surface	Mannan
	Mononuclear phagocytes		Glucuronoxylomannan
	DCs		Glycoinositolphospholipids
	T-Lymphocytes		Envelope proteins
			Heat-shock protein 60,70
			Fibrinogen
TLR5	PMNs	Cell surface	Flagellin
	Mononuclear phagocytes		
	DCs		
TLR6	T-Lymphocytes	Cell surface	Diacyl lipopeptides
	B-Lymphocytes		lipoteichoic acid
	Mononuclear phagocytes		Zymosan
	DCs		
TLR7	T-Lymphocytes	Endolysosome	ssRNA
	B-Lymphocytes		Imidazoquinoline
	DCs		
TLR8	T-Lymphocytes	Endolysosome	Loxoribine
	Mononuclear phagocytes		ssRNA
	DCs		Imidazoquinoline
TLR9	T-Lymphocytes	Endolysosome	Bropirimin
	B-Lymphocytes		DNA
	Mononuclear phagocytes		CpG-DNA
	DCs		Hemozoin
TLR10	B-Lymphocytes	Cell surface	not determined
	DCs		
TLR11		Cell surface	Profilin-like molecule
			not determined
TLR12			not determined
TLR13			not determined
